# A metabolomic approach to elucidate the inhibitory effects of baicalin in pulmonary fibrosis

**DOI:** 10.1080/13880209.2021.1950192

**Published:** 2021-08-06

**Authors:** Hong Chang, Hong-yu Meng, Wan-fu Bai, Qing-gang Meng

**Affiliations:** a Department of Pharmacy, Baotou Medical College, Baotou, China; b Nephroendocrine Department, Dongzhimen Hospital, Beijing University of Chinese Medicine, Beijing, China; c Department of Traditional Chinese Medicine, Beijing University of Chinese Medicine, Beijing, China

**Keywords:** Traditional Chinese medicine, serum metabolomics, UPLC-QTOF/MS, mechanism research

## Abstract

**Context:**

Baicalin, a major flavonoid extracted from *Scutellaria baicalensis* Georgi (Lamiaceae), has been shown to exert therapeutic effects on pulmonary fibrosis (PF).

**Objective:**

To use serum metabolomics combined with biochemical and histopathological analyses to clarify anti-PF mechanisms of baicalin on metabolic pathways and the levels of potential biomarkers.

**Materials and methods:**

Forty male Sprague–Dawley rats were randomly divided into the control, PF model, prednisolone acetate-treated (4.2 mg/kg/day) and baicalin-treated (25 and 100 mg/kg/day) groups. A rat model of PF was established using a tracheal injection of bleomycin, and the respective drugs were administered intragastrically for 4 weeks. Histomorphology of lung tissue was examined after H&E and Masson’s trichrome staining. Biochemical indicators including SOD, MDA and HYP were measured. Serum-metabonomic analysis based on UPLC-Q-TOF/MS was used to clarify the changes in potential biomarkers among different groups of PF rats.

**Results:**

Both doses of baicalin effectively alleviated bleomycin-induced pathological changes, and increased the levels of SOD (from 69.48 to 99.50 and 112.30, respectively), reduced the levels of MDA (from 10.91 to 5.0 and 7.53, respectively) and HYP (from 0.63 to 0.41 and 0.49, respectively). Forty-eight potential biomarkers associated with PF were identified. Meanwhile, the metabolic profiles and fluctuating metabolite levels were normalized or partially reversed after baicalin treatment. Furthermore, baicalin was found to improve PF potentially by the regulation of four key biomarkers involving taurine and hypotaurine metabolism, glutathione metabolism, and glycerophospholipid metabolism.

**Conclusions:**

These findings revealed the anti-fibrotic mechanisms of baicalin and it may be considered as an effective therapy for PF.

## Introduction

Pulmonary fibrosis (PF) is a chronic, inexorably progressive, scarring interstitial lung disorder characterized by fibroblast proliferation and excessive accumulation of extracellular matrix (ECM) proteins (Wuyts et al. 2013). PF can lead to a series of severe manifestations, such as distorted lung architecture, declining lung function, and ultimately respiratory failure (White et al. 2016). The incidence of PF is increasing worldwide, and it has been shown to be a major contributor to mortality among coronavirus disease 2019 (COVID-19) patients, posing a serious threat to human health (Chen et al. 2020; George et al. 2020).

At present, few effective treatments are available (Jo et al. 2018). The current clinical therapeutics for pulmonary fibrosis primarily rely on anti-inflammatory and immunosuppressive drugs, such as glucocorticoids, to control the symptoms, but they appear to neither halt the progression of fibrosis nor improve life expectancy. Moreover, high-dosage and/or long-term use of glucocorticoids can lead to serious adverse effects (Hu et al. 2015). However, the pathogenesis of PF is a complex process involving multiple factors, including inflammation, immune mechanisms, epithelial-mesenchymal transition (EMT), and oxidative stress. Furthermore, these factors can interact to stimulate the development and progression of PF (Srour and Thébaud 2015). Therefore, treatments that target multiple pathways in PF may offer a helpful resolution to this debilitating disease.

Traditional Chinese medicine (TCM) has made considerable progress in preventing or treating a variety of complicated diseases, including liver, kidney, and lung fibrotic diseases, via multitarget and whole-body regulation (Song et al. 2016; Zhang et al. 2018b; Chang et al. 2020). Many active ingredients in TCM, such as triptolide (Chen et al. 2018), berberine (Chitra et al. 2013), andrographolide (Yan et al. 2018), and the total aglycone extracts of *Scutellaria baicalensis* Georgi (Lamiaceae), (Fang et al. 2016), have shown promise in pharmacodynamic studies. Among them, flavonoids have attracted considerable attention and have shown potential as effective antifibrotic compounds due to a wide range of pharmacological activities, including anti-inflammatory, antioxidative, antibacterial, antiviral, and antitumor functions (Hu et al. 2015). However, effective evaluation and in-depth analyses of the mechanisms of their effects are still urgently needed.

Metabolomics is a holistic approach that is consistent with natural herbal medicine theory and thus provides a powerful method for assessing the efficacy and mechanisms of TCM (Wang et al. 2011). Recently, several remarkable achievements have been made in antifibrotic drug discovery and development by using metabolomics (Cai et al. 2018; Chang et al. 2020). The dried roots of the medicinal plant *S. baicalensis*, called Huangqin in China, have been used to treat lung infections, liver problems, inflammation, diarrhoea, and dysentery for thousands of years (Zhao et al. 2016). The flavonoid baicalin is a major bioactive compound derived from *S. baicalensis* and has been shown to have various pharmacological effects in the lungs and liver, such as antioxidant, anti-inflammatory, anticancer, and antifibrotic activities (Liu et al. 2015; Lu et al. 2017; Wu et al. 2018; Zhang et al. 2018a). Recent reports have shown that baicalin exerts therapeutic effects in rats with bleomycin-induced lung fibrosis, a widely used model that resembles human PF both histologically and physiologically (Della et al. 2015), by regulating the transforming growth factor (TGF)-β1 pathway and hepatic lipid levels (Huang et al. 2016). However, to the best of our knowledge, there are few reports explaining its potential antifibrotic mechanisms using multichannel holistic approaches.

In the present study, we used serum metabolomics combined with biochemical and histopathological analyses to evaluate the therapeutic effect of baicalin on PF. This approach was expected to elucidate anti-PF mechanisms by analyzing the impact of baicalin on the levels of potential biomarkers and on metabolic pathways.

## Materials and methods

### Chemicals and reagents

Baicalin (purity > 85%) was supplied by Xi’an Watson Biotechnology Co., Ltd. (Xi’an, China). Bleomycin hydrochloride was obtained from Nippon Kayaku Co. (Tokyo, Japan) and was dissolved in saline (2.5 mg in 1 mL of saline). Prednisolone acetate was purchased from Zhejiang Xianju Pharmaceutical Co., Ltd. (Zhejiang, China). HYP, MDA, and SOD assay kits and Masson’s trichrome staining kits were obtained from Nanjing Jiancheng Bioengineering Institute (Nanjing, China). Methanol, acetonitrile and formic acid (HPLC grade) were purchased from Thermo Fisher Scientific (Waltham, MA, USA). Leucine enkephalin was purchased from Sigma-Aldrich (St. Louis, MO, USA). Deionised water was produced using a Milli-Q^®^ Ultrapure Water System (Millipore, Billerica, USA).

### Animal model and groups

Forty male Sprague–Dawley rats (170–200 g, 6 weeks old) were obtained from the Department of Experimental Animal Sciences, Peking University Health Science Centre (Beijing, China). Experimental rats were reared at 23–25 °C with a relative humidity of 45–50%, and 12 h light/dark cycle. After an acclimation period of one week, the rats were randomly divided into the control group (C group, *n* = 8), model group (M group, *n* = 8), baicalin high-dose group (Bai-h group, *n* = 8), baicalin low-dose group (Bai-l group, *n* = 8), and positive drug group (Y group, *n* = 8). A model of PF was established by intratracheal instillation of bleomycin (Huang et al. 2016). Under pentobarbital sodium anaesthesia, the rats in the M, Bai-h, Bai-l, and Y groups were injected with 5 mg/kg (approximately 0.2 mL) bleomycin (Hospira, Lake Forest, IL, USA) through the cartilaginous rings of the trachea. Rats in the Bai-l and Bai-h groups were treated with baicalin (25 and 100 mg/kg/day, respectively), and those in the Y group were treated with prednisolone acetate (4.2 mg/kg/day). The dosage of medication given to the rats was calculated according to the conversion of animal dose to human equivalent doses based on body surface area (Nair and Jacob 2016). Rats in the C and M groups were treated with an equal volume of physiological saline (5 mL/kg/day). All treatments were administered by gavage for 4 weeks, starting day one after bleomycin instillation. Rats were given access to water and food (standard rodent diet) *ad libitum*. This animal experiment was approved by the Animal Experiment Ethics Committee of Baotou Medical College, Baotou, Inner Mongolia, China.

### Sample collection and preparation

At the end of the experiment, all rats were anaesthetised using sodium pentobarbital (35 mg/kg, intraperitoneal injection), and blood samples were collected via the abdominal aorta. Then, the serum was separated by centrifugation at 3000 rpm for 15 min at 4 °C and stored at −80 °C. A section of lung tissue was harvested from each rat, fixed with 10% paraformaldehyde, and embedded in paraffin. The remaining lung tissues were stored at −80 °C until hydroxyproline (HYP) analysis.

### Histological examination

Paraffin-embedded lung tissues were sliced into 5 µm sections, and haematoxylin and eosin (H&E) and Masson’s trichrome staining were used to evaluate PF. Fibrosis was scored in 10 randomly selected nonoverlapping fields per rat on a scale of 0–4 according to the procedure described by Szapiel et al. (1979).

### Biochemical indicator measurements

The serum levels of superoxide dismutase (SOD) and malondialdehyde (MDA) were measured according to the instruction manual provided with the kit. The HYP content in the tissue samples was used to assess the lung collagen content and was determined per the manufacturer’s instructions.

### UPLC-QTOF/MS analysis

In each group, six serum samples were randomly selected for ultra-performance liquid chromatography quadrupole time of flight mass spectrometry (UPLC-QTOF/MS) analysis. Detailed liquid phase and mass spectrometry parameter descriptions were as follow:

UHPLC conditions: Waters Acquity™UHPLC (consisting of a vacuum degasser, autosampler, binary pump, photodiode array detector, and oven) was equipped with an ACQUITY UPLC^®^ BEH C18 column (2.1 mm × 50 mm, i.d. 1.7 µm, Waters Corp). The analytical column was maintained at a temperature of 40 °C and the mobile phase was composed of acetonitrile (A) and water (B), each containing 0.1% formic acid. The gradient for the serum sample was used: 2–100% A for 15 min. The injection volume was 2 µL with a flow rate of 0.4 mL/min. The eluent was introduced to the mass spectrometer directly.

MS analysis was performed on a Q-TOF analyzer in the SYNAPTHDMS system (Waters Corporation) in the positive-ion (ESI^+^) and negative-ion (ESI^−^) mode, using the following parameters: capillary voltage, 1300 V (ESI^+^), 1500 V (ESI^−^); sample cone voltage, 60 V (ESI^+^), 770 V (ESI^−^); source temperature, 110 °C; desolvation temperature, 350 °C; desolvation gas flow, 750 L/h; cone gas flow, 20 L/h. MS data were collected in the full scan mode from *m/z* 100–1500. All the data were acquired using an independent reference lock mass via the LockSpray™ interface to ensure accuracy and reproducibility during the MS analysis. Leucine encephalin was used as the reference ion ([M + H]^+^ = 556.2771) and [M − H]^−^ = 554.2615) at a concentration of 1 ng/mL under a flow rate of 30 µL/min. The data were collected in the centroid mode, and the LockSpray frequency was set at 15 s and was averaged over five scans for correction.

### Data analysis

Data were acquired and processed using MassLynx V4.1 and MarkerLynx software (Waters Corp., Milford, USA). Multivariate data analysis was conducted using EZinfo software (Waters Corp.). Supervised partial least squares-discriminant analysis (PLS-DA) was used to visualise general clustering and trends among the samples. Orthogonal projections to latent structures discriminant analysis (OPLS-DA) were used to compare samples between the M and C groups. Model quality was assessed using the goodness-of-fit (R^2^Y) and predictive ability (Q^2^) parameters. R^2^Y and Q^2^ values close to 1.0 represented an excellent model. The s-plot constructed from the loading plots of OPLS-DA was used to screen the differential metabolites, and differences were determined using variable importance in projection (VIP) value >1 and *p* < 0.05 (*p*-values from two-tailed Student’s *t*-test). The endogenous differential metabolites were identified on the basis of exact molecular mass, MS/MS fragments, mass spectra, and chromatographic retention times obtained by searching online databases. Exact molecular mass data were confirmed from redundant *m/z* peaks. Moreover, the mass tolerance between the measured *m/z* value and the exact mass of the component of interest was set to within 5 mDa.

Heatmap construction and metabolomics pathway analysis (MetPA) of the identified differential metabolites were performed using MetaboAnalyst software 4.0. The receiver operating characteristic (ROC) curves were constructed using ggplot2 to determine the diagnostic effectiveness of the selected critical biomarkers, and the classification performance (1-specificity and sensitivity with the highest accuracy) was assessed according to the area under the curve (AUC) values of the ROC curves. Network analysis of the identified biomarkers was carried out based on the Kyoto Encyclopaedia of Genes and Genomes (KEGG) pathway database. Statistical analyses were performed using IBM SPSS Statistics 23. A one-factor ANOVA followed by Tukeys *post hoc* test was used for group comparisons, and between-group differences were analyzed using Student’s unpaired *t*-test. *p*-Values less than 0.05 were considered statistically significant. All data are presented as mean ± SD.

## Results

### Histopathology and hydroxyproline analyses

**Figure 1.**
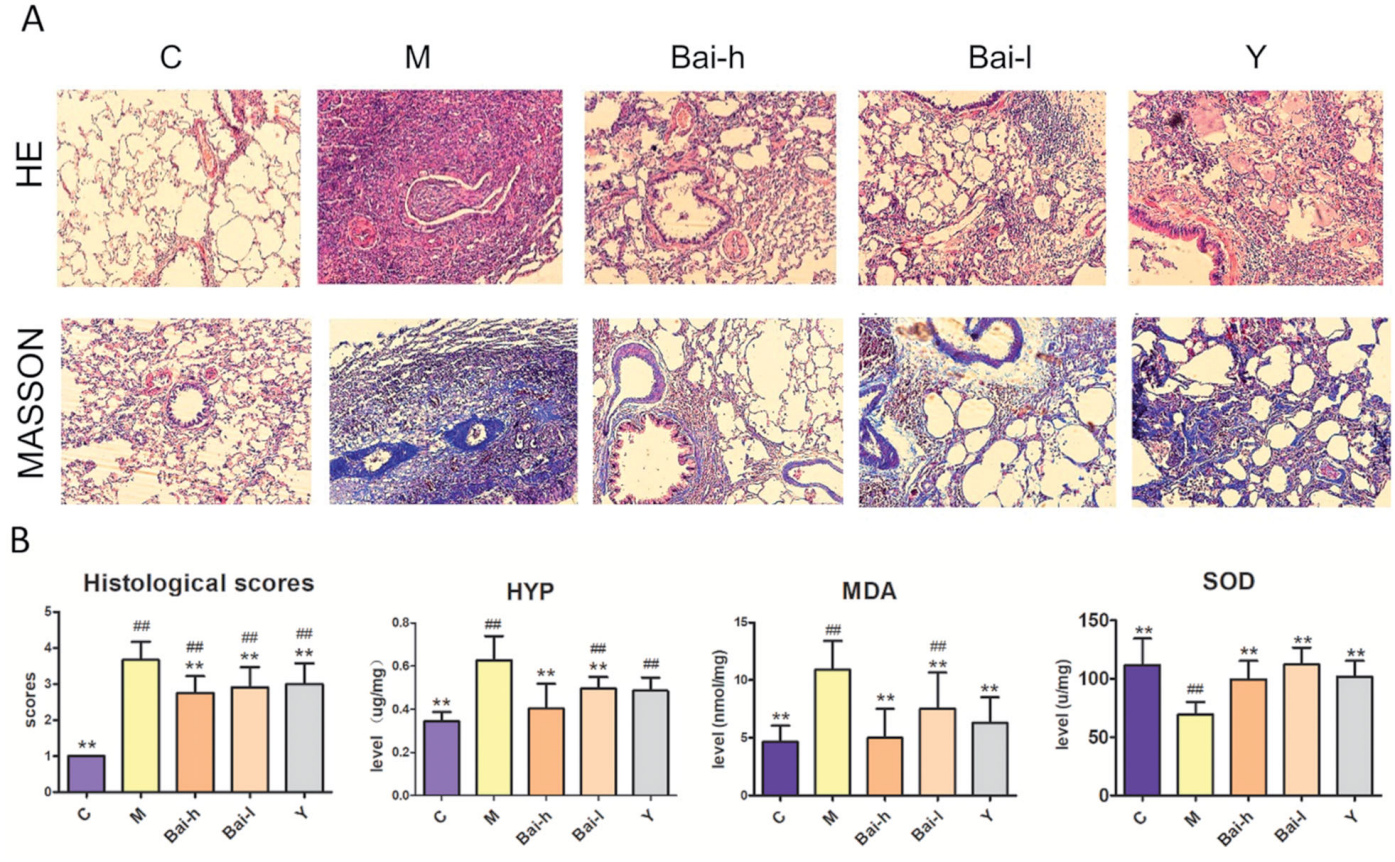
The pathological analysis of lung sections and determination of biochemical indicators in all groups. (A) Representative photomicrographs of the H&E staining and Masson staining (Images magnification: all picture 200×). (B) Result of HYP, SOD, MDA and mean histological scores. The degree of severity was assigned the following scores: 0, no evidence of fibrosis; 1, focal regions of fibrosis involving less than 20% of the lung; 2, 20–50% of the lung and fibrotic regions; 3, 51–75% of the lung and fibrotic regions; and 4, focal regions of fibrosis involving more than 75% of the lung. All values represent the mean ± SD. Statistical significance was calculated with ANOVA. ^#^*p* < 0.05, ^##^*p* < 0.01 compared to the control group; *p < 0.05, ** *p* < 0.01 compared to the model group.

Representative photomicrographs of haematoxylin and eosin (H&E) and Masson’s trichrome staining of the lung tissue are shown in Figure 1(A). H&E-stained lung tissue from the control (C) group showed clear and intact structures, thin alveolar septa, no inflammatory cell infiltration, and no accumulation of collagen fibres. In contrast, the model (M) group exhibited damaged and fused alveolar structures, the presence of a large number of fibroblasts, and diffuse fibrosis. This altered histology was significantly improved in all treatment groups. Masson’s trichrome stains collagenous ECM blue, which was used to confirm the effects on collagen deposition.

The model group showed a large area of blue collagen fibres. This staining was decreased significantly in each treatment group, remaining only around the bronchial wall. Parts of the lung exhibited normal alveolar structures, especially in the Bai-h (100 mg/kg/day) treatment group. The lung collagen fibres were graded according to the area of staining. The results showed that baicalin treatment effectively alleviated bleomycin-induced pathological changes. In addition, HYP content, a marker of collagen deposition, corroborated the antifibrotic activity of baicalin. Compared with that of the control group, the HYP level was significantly increased (*p* < 0.05) in the model group and was attenuated in both the Bai-h and Bai-l groups. There was no significant difference in HYP levels between the high-dose baicalin and control groups.

### Determination of biochemical indicators of oxidative stress

MDA is an index of lipid oxidation, while SOD is an antioxidant enzyme. Both were assayed in all groups to evaluate the antioxidant activity of baicalin (as shown in Figure 1(B) and Table 1). Compared with those in the control group, the levels of MDA and SOD were significantly increased and decreased, respectively, in the bleomycin-exposed rats (*p* < 0.01), thus indicating the presence of oxidative stress in this model. After treatment with Bai-h or Bai-l, the levels of MDA and SOD were ameliorated (*p* < 0.01). It is speculated that baicalin plays an antioxidant role in attenuating the lung fibrosis induced by bleomycin.

**Table 1.** The analysis results from determination of SOD, MDA, HYP and histlogical scores (mean ± SD).

Group	Serum SOD (µg/mg)	Serum MDA (nmol/mg)	Tissue HYP (µg/mg)	Histlogical scores
C	111.40 ± 22.95**	4.63 ± 1.45**	0.34 ± 0.04**	1.00 ± 0.00**
M	69.48 ± 10.60^##^	10.91 ± 2.50^##^	0.63 ± 0.11^##^	3.67 ± 0.50^#^
Bai-h	99.50 ± 15.69**	5.00 ± 2.53**	0.41 ± 0.11**	2.75 ± 0.46^##^**
Bai-l	112.30 ± 14.25**	7.53 ± 3.13^##^**	0.49 ± 0.05^##^**	2.90 ± 0.57^##^**
Y	101.52 ± 13.79**	6.29 ± 2.24**	0.49 ± 0.06^##^	3.00 ± 0.58^##^**

^#^*p* < 0.05, ^##^*p* < 0.01 compared to control group; *p < 0.05, **p < 0.01 compared to model group.

### Metabolic profile analysis

**Figure 2.**
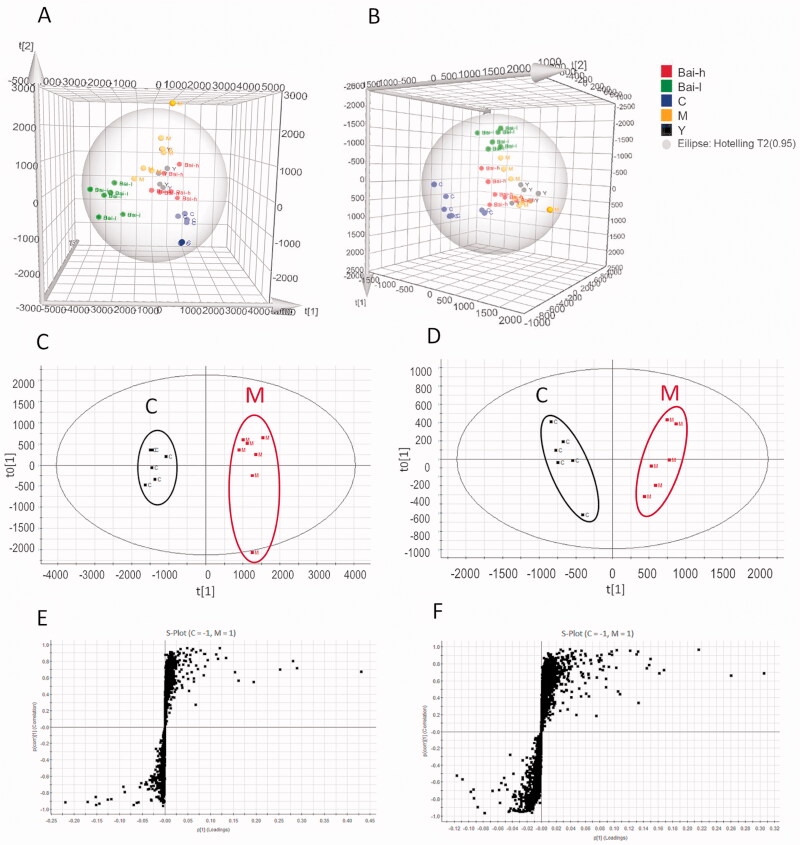
The PLS-DA score plots of serum samples analysed in positive (A) and negative (B) ion models from all groups; OPLS‑DA score plots of serum samples analysed in positive (C) and negative (D) ion models comparing pulmonary fibrosis metabolite profiles from the control to those from the model group; S-plots constructed from the OPLS-DA of serum samples in positive (E) and negative (F) ion models.

Serum metabolic profiling was performed using UPLC-QTOF/MS in positive and negative ion modes. The metabolic alteration analysis of PLS-DA score plots for all groups showed good clustering and clear separation in both positive and negative ion modes (Figure 2(A,B)). The model group was significantly divided from the control group, indicating that the PF model was successfully established. Furthermore, the positive drug (prednisolone acetate) and high- and low-dose baicalin groups were located between the model and control groups, indicating the positive regulatory effect of baicalin on endogenous metabolites in the serum of rats with PF. The serum parameters exhibited good fitness and predictive values in both the positive (R^2^Y = 96%, Q^2^ (cum) = 83%) and negative (R^2^Y = 83%, Q^2^ (cum) = 55%) ion modes.

### Identification and quantification of potential biomarkers

**Figure 3.**
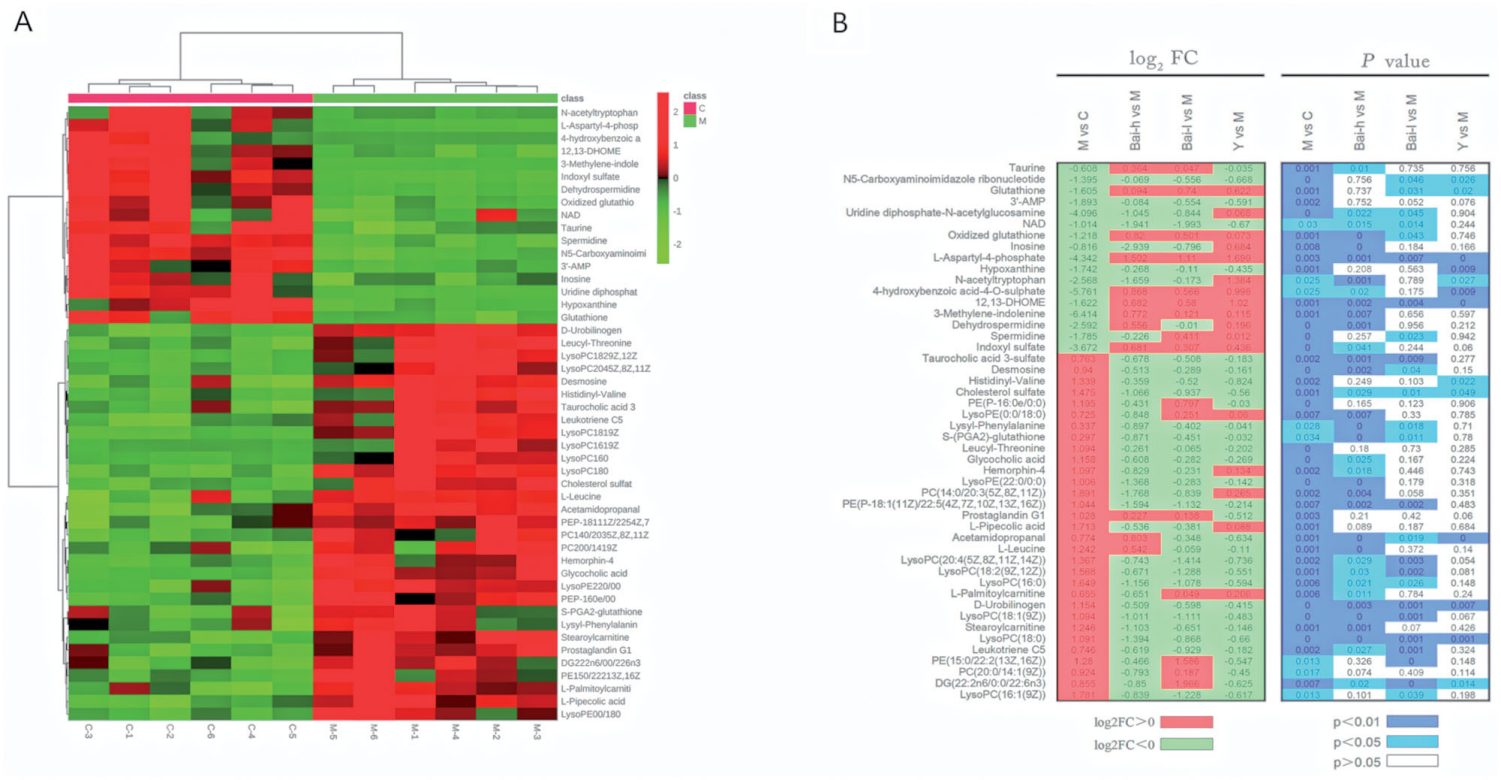
Analysis of the identified differential metabolites. (A) Heatmap of the differential abundance of metabolites in the control and model groups. (B) Heatmap of the differential abundance expression levels and associated *p*-values for the control, low and high doses of baicalin vs model groups. Statistical significance was calculated with Student’s *t*-test.

Identified metabolites were selected by using OPLS-DA of the control and model groups (Figure 2(C,D)) according to their VIP value in the S-plots (Figure 2(E,F)). Finally, in total, 25 and 23 differential metabolites in the positive and negative ion modes, respectively, were selected based on VIP >1.0 and *p* < 0.05. The fold change (FC) and log_2_(FC) of each identified metabolite between the control and model groups were also calculated; details are listed in Table 2. The mathematical models established in the positive (R^2^Y = 98%, Q^2^ (cum) = 95%) and negative (R^2^Y = 95%, Q^2^ (cum) = 88%) ion modes showed a good degree of fit and predictive ability, with R^2^Y and Q^2^ values close to 100%. Furthermore, hierarchical clustering was performed for the sets of these differentially abundant metabolites, and the results are presented as a heatmap (Figure 3(A)). 31 metabolites were increased (red) and 17 were decreased (green) in the model group compared with the control group.

**Table 2.** The results of the identified differential metabolites from the control and model groups.

*m/z*	*t_R_* (min)		VIP	HMDB ID	Formula	Compound	Passway	*p*-Value (*t*-test)	Trend (M vs C)	Fold change (M vs C)	Log_2_(FC) (M vs C)
Positive ion model											
496.34		10.87	23.61	HMDB10382	C_24_H_50_NO_7_P	LysoPC (16:0)	Glycerophospholipid metabolism;	0.006	↑	3.136	1.649
782.57		12.94	15.27	HMDB08263	C_42_H_82_NO_8_P	PC (20:0/14:1 (9Z))	Glycerophospholipid metabolism;	0.017	↑	1.898	0.924
524.37		12.34	14.97	HMDB10384	C_26_H_54_NO_7_P	LysoPC (18:0)	Glycerophospholipid metabolism;	0.000	↑	2.130	1.091
758.57		12.84	12.61	HMDB08909	C_42_H_80_NO_8_P	PE (15:0/22:2 (13Z,16Z))	Glycerophospholipid metabolism;	0.013	↑	2.428	1.280
128.14		12.85	8.05	HMDB01257	C_7_H_19_N_3_	Spermidine	Glutathione metabolism;	0.000	↓	0.290	−1.785
152.05		11.81	7.92	HMDB11664	C_9_H_7_N	3-Methylene-indolenine	Tryptophan metabolism	0.001	↓	0.012	−6.414
703.57		12.98	7.22	HMDB56350	C_47_H_76_O_5_	DG (22:2n6/0:0/22:6n3)	Unidentified	0.007	↑	1.809	0.855
522.36		11.20	5.14	HMDB02815	C_26_H_52_NO_7_P	LysoPC (18:1 (9Z))	Glycerophospholipid metabolism;	0.000	↑	2.135	1.094
520.34		10.29	4.92	HMDB10386	C_26_H_50_NO_7_P	LysoPC (18:2 (9Z,12Z))	Glycerophospholipid metabolism;	0.001	↑	2.964	1.568
544.34		10.28	3.94	HMDB10395	C_28_H_50_NO_7_P	LysoPC (20:4 (5Z,8Z,11Z,14Z))	Glycerophospholipid metabolism;	0.002	↑	2.579	1.367
494.32		9.91	3.50	HMDB10383	C_24_H_48_NO_7_P	LysoPC (16:1 (9Z))	Glycerophospholipid metabolism;	0.013	↑	3.436	1.781
196.02		2.52	3.25	HMDB00682	C_8_H_7_NO_4_S	Indoxyl sulphate	Unidentified	0.000	↓	0.078	−3.672
137.05		0.96	2.83	HMDB00157	C_5_H_4_N_4_O	Hypoxanthine	Purine metabolism;	0.001	↓	0.299	−1.742
573.31		11.04	2.22	HMDB04158	C_33_H_42_N_4_O_6_	d-Urobilinogen	Porphyrin and chlorophyll metabolism	0.000	↑	2.225	1.154
200.99		1.49	2.14	HMDB59982	C_7_H_6_O_6_S	4-hydroxybenzoic acid-4-O-sulphate	Unidentified	0.025	↓	0.018	−5.761
400.34		10.92	2.08	HMDB00222	C_23_H_45_NO_4_	l-Palmitoyl carnitine	Unidentified	0.006	↑	1.575	0.655
132.10		1.36	1.93	HMDB00687	C_6_H_13_NO_2_	l-Leucine	Valine, leucine and isoleucine biosynthesis	0.001	↑	2.365	1.242
126.13		12.51	1.58	HMDB12925	C_7_H_17_N_3_	Dehydro spermidine	Arginine and proline metabolism	0.000	↓	0.166	−2.592
214.00		0.87	1.44	HMDB12250	C_4_H_8_NO_7_P	l-Aspartyl-4-phosphate	Biosynthesis of amino acids	0.003	↓	0.049	−4.342
269.09		1.37	1.31	HMDB13713	C_13_H_14_N_2_O_3_	N-acetyltryptophan	Unidentified	0.025	↓	0.169	−2.568
646.28		12.35	1.26	HMDB12993	C_30_H_45_N_3_O_9_S	Leukotriene C5	Unidentified	0.002	↑	1.677	0.746
297.25		11.68	1.21	HMDB04705	C_18_H_34_O_4_	12,13-DHOME	Linoleic acid metabolism	0.001	↓	0.325	−1.622
116.07		0.70	1.18	HMDB12880	C_5_H_9_NO_2_	Acetamido propanal	Arginine and proline metabolism	0.001	↑	1.710	0.774
112.09		0.58	1.14	HMDB00716	C_6_H_11_NO_2_	l-Pipecolic acid	Lysine degradation;	0.001	↑	3.278	1.713
428.37		11.92	1.11	HMDB00848	C_25_H_49_NO_4_	Stearoyl carnitine	Unidentified	0.001	↑	2.371	1.246
Negative ion model											
436.28		11.24	13.61	HMDB11152	C_21_H_44_NO_6_P	PE (P-16:0e/0:0)	Glycerophospholipid metabolism;	0.000	↑	2.289	1.195
480.31		12.35	8.83	HMDB11129	C_23_H_48_NO_7_P	LysoPE (0:0/18:0)	Glycerophospholipid metabolism;	0.007	↑	1.653	0.725
464.31		12.74	8.47	HMDB00138	C_26_H_43_NO_6_	Glycocholic acid	Primary bile acid biosynthesis;	0.000	↑	2.232	1.158
571.29		11.14	6.33	HMDB00572	C_24_H_40_N_5_O_8_+	Desmosine	Unidentified	0.000	↑	1.918	0.940
606.07		1.07	5.77	HMDB00290	C_17_H_27_N_3_O_17_P_2_	Uridine diphosphate-N -acetylglucosamine	Amino sugar and nucleotide sugar metabolism	0.000	↓	0.058	−4.096
800.54		12.86	5.76	HMDB07881	C_42_H_78_NO_8_P	PC (14:0/20:3 (5Z,8Z,11Z))	Glycerophospholipid metabolism	0.002	↑	3.708	1.891
611.14		1.21	5.52	HMDB03337	C_20_H_32_N_6_O_12_S_2_	Oxidised glutathione	Glutathione metabolism;	0.001	↓	0.430	−1.218
124.01		0.66	4.84	HMDB00251	C_2_H_7_NO_3_S	Taurine	Primary bile acid biosynthesis	0.001	↓	0.656	−0.608
507.27		11.17	4.20	HMDB28898	C_11_H_18_N_4_O_3_	Histidinyl-Valine	Unidentified	0.002	↑	2.529	1.339
306.08		0.96	4.07	HMDB00125	C10H17N3O6S	Glutathione	Glutathione metabolism	0.001	↓	0.329	−1.605
640.29		12.38	4.04	HMDB13062	C_30_H_47_N_3_O_10_S	S-(PGA2)-glutathione	Unidentified	0.034	↑	1.228	0.297
564.24		12.74	3.95	HMDB59788	C_29_H_35_N_5_O_7_	Hemorphin-4	Unidentified	0.002	↑	2.138	1.097
640.26		10.91	3.95	HMDB02581	C_26_H_45_NO_10_S_2_	Taurocholic acid 3-sulphate	Unidentified	0.002	↑	1.697	0.763
369.23		5.64	3.78	HMDB13039	C_20_H_34_O_6_	Prostaglandin G1	Unidentified	0.003	↑	2.039	1.028
582.38		12.86	3.47	HMDB11520	C_27_H_56_NO_7_P	LysoPE (22:0/0:0)	Glycerophospholipid metabolism;	0.000	↑	2.008	1.006
774.53		12.88	3.02	HMDB11425	C_45_H_78_NO_7_P	PE (P-18:1 (11Z)/22:5 (4Z,7Z,10Z,13Z,16Z))	Glycerophospholipid metabolism;	0.007	↑	2.061	1.044
509.29		12.55	3.01	HMDB28939	C_10_H_20_N_2_O_4_	Leucyl-Threonine	Unidentified	0.000	↑	2.134	1.094
313.08		1.38	2.91	HMDB00195	C_10_H_12_N_4_O_5_	Inosine	Purine metabolism;	0.008	↓	0.568	−0.816
384.04		0.79	2.64	HMDB12268	C_9_H_14_N_3_O_9_P	N5-Carboxyaminoimidazole ribonucleotide	Unidentified	0.000	↓	0.380	−1.395
709.12		1.09	2.59	HMDB00902	C_21_H_28_N_7_O_14_P_2_+	NAD (Nicotinamide adenine dinucleotide)	Nicotinate and nicotinamide metabolism	0.030	↓	0.495	−1.014
585.35		12.38	2.25	HMDB28958	C_15_H_23_N_3_O_3_	Lysyl-Phenylalanine	Unidentified	0.028	↑	1.263	0.337
346.06		1.00	2.12	HMDB03540	C_10_H_14_N_5_O_7_P	3′-AMP	Purine metabolism;	0.002	↓	0.269	−1.893
931.62		11.21	2.04	HMDB00653	C_27_H_46_O_4_S	Cholesterol sulphate	Steroid hormone biosynthesis	0.001	↑	2.780	1.475

Additionally, to explore the effect of baicalin on the concentrations of these 48 metabolites, their means between the drug-treatment and model groups were compared using Student’s *t*-tests. The results for the log_2_(FC) and *p*-value heatmap (Figure 3(B)) showed that 29, 20, and 11 biomarkers were reversed by treatment with high- and low-dose baicalin and prednisolone acetate, respectively.

### Metabolic pathway analysis and the selection of key biomarkers

**Figure 4.**
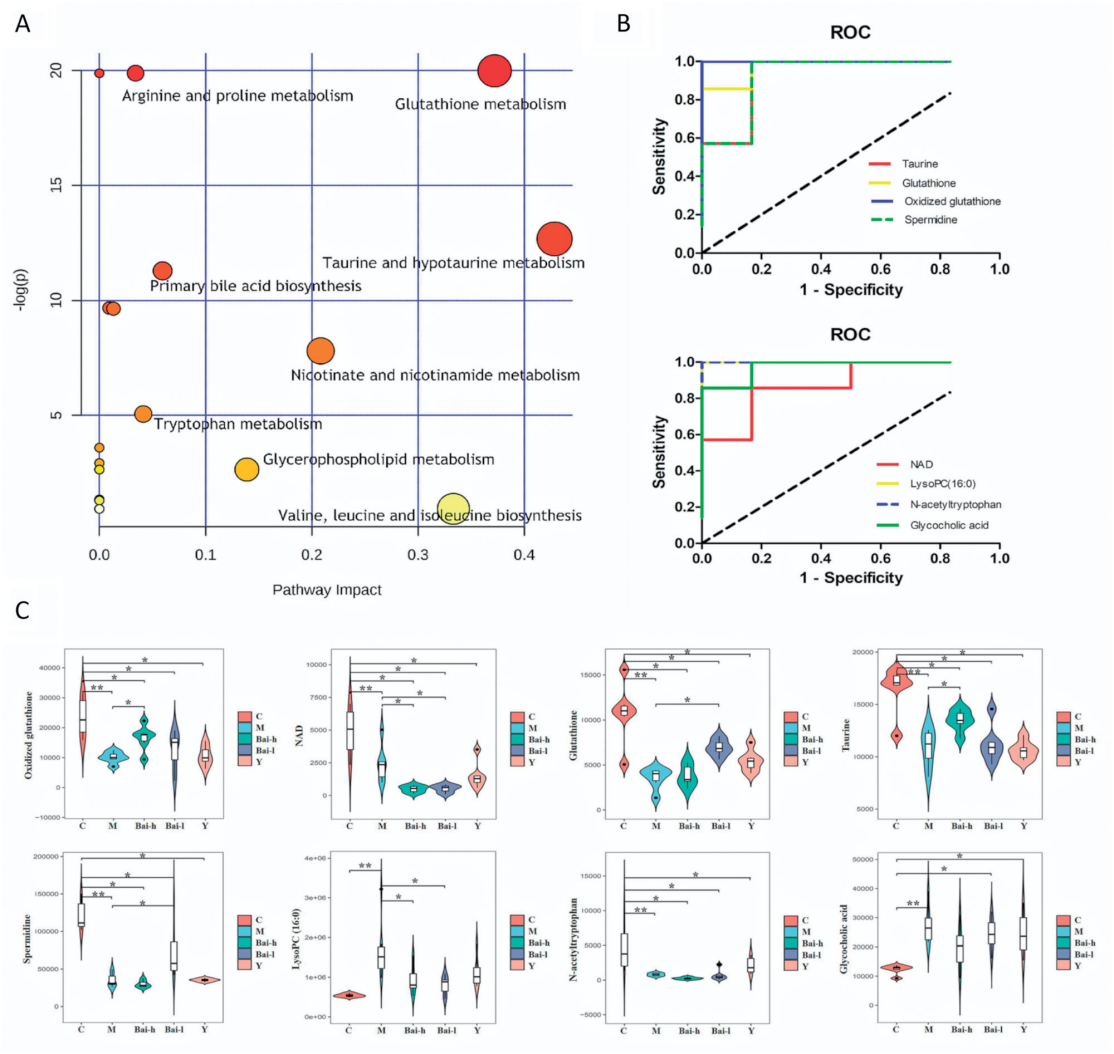
Analysis of related metabolic pathways and crucial biomarkers. (A) Metabolism pathway analysis of serum samples with MetPA, as analyzed by MetaboAnalyst (http://www.metaboanalyst.ca/MetaboAnalyst/). The size and colour of each circle were based on the pathway impact value and *p*-value, respectively. (B) ROC curves of the diagnosis of RF based on the selected crucial biomarkers. AUC, area under the curve. (C) Change trends of eight key biomarker relative intensities in different groups for pulmonary fibrosis. *p < 0.05, **p < 0.01.

To understand the metabolic pathways associated with the 48 differentially abundant metabolites and to further select critical biomarkers, MetaboAnalyst tools were used to map these metabolites into pathways (Figure 4(A)). The results revealed that seven metabolic pathways involved in taurine and hypotaurine metabolism, glutathione metabolism, nicotinate, and nicotinamide metabolism, glycerophospholipid metabolism, primary bile acid biosynthesis, tryptophan metabolism, and arginine and proline metabolism were obviously dysregulated (−Log(*p*) > 2 and impact >0.02) in bleomycin-exposed rats. Finally, eight serum metabolites were identified from these selected metabolic pathways. These were considered key biomarkers associated with the mechanisms underlying PF progression and are potential biomarkers for monitoring PF.

### Diagnostic potential of key metabolites

The diagnostic ability of the abovementioned eight key biomarkers was evaluated using ROC curves (Figure 4(B)). The results showed that the eight biomarkers had AUC values greater than 0.85 and were top-ranked candidates expected to become biomarkers for the diagnosis of PF and objective indices for the evaluation of drug efficacy and prognosis.

To further explain the mechanism of baicalin-mediated protection against PF, the relative concentrations of the eight crucial biomarkers were calculated in all groups and are shown in Figure 4(C). Compared with that in the model group, low-dose baicalin significantly increased the contents of glutathione and spermidine (*p* < 0.05). High-dose baicalin significantly increased the contents of oxidized glutathione and taurine (*p* < 0.05). The content of lysophosphatidylcholine (16:0) [LysoPC (16:0)] was restored to normal levels by both high and low doses of baicalin (*p* < 0.05).

## Discussion

The results of serum metabolomic studies on rats with PF showed that a variety of metabolites and multiple metabolic pathways were altered in the model group after 4 weeks. According to MetPA, the selected potential biomarkers are mainly involved in taurine and hypotaurine metabolism, glutathione metabolism, nicotinate and nicotinamide metabolism, glycerophospholipid metabolism, primary bile acid biosynthesis, tryptophan metabolism, and arginine and proline metabolism. The differential metabolites corresponding to these metabolic pathways were upregulated or downregulated at different stages of disease development, suggesting their key roles in disease evolution.

**Figure 5.**
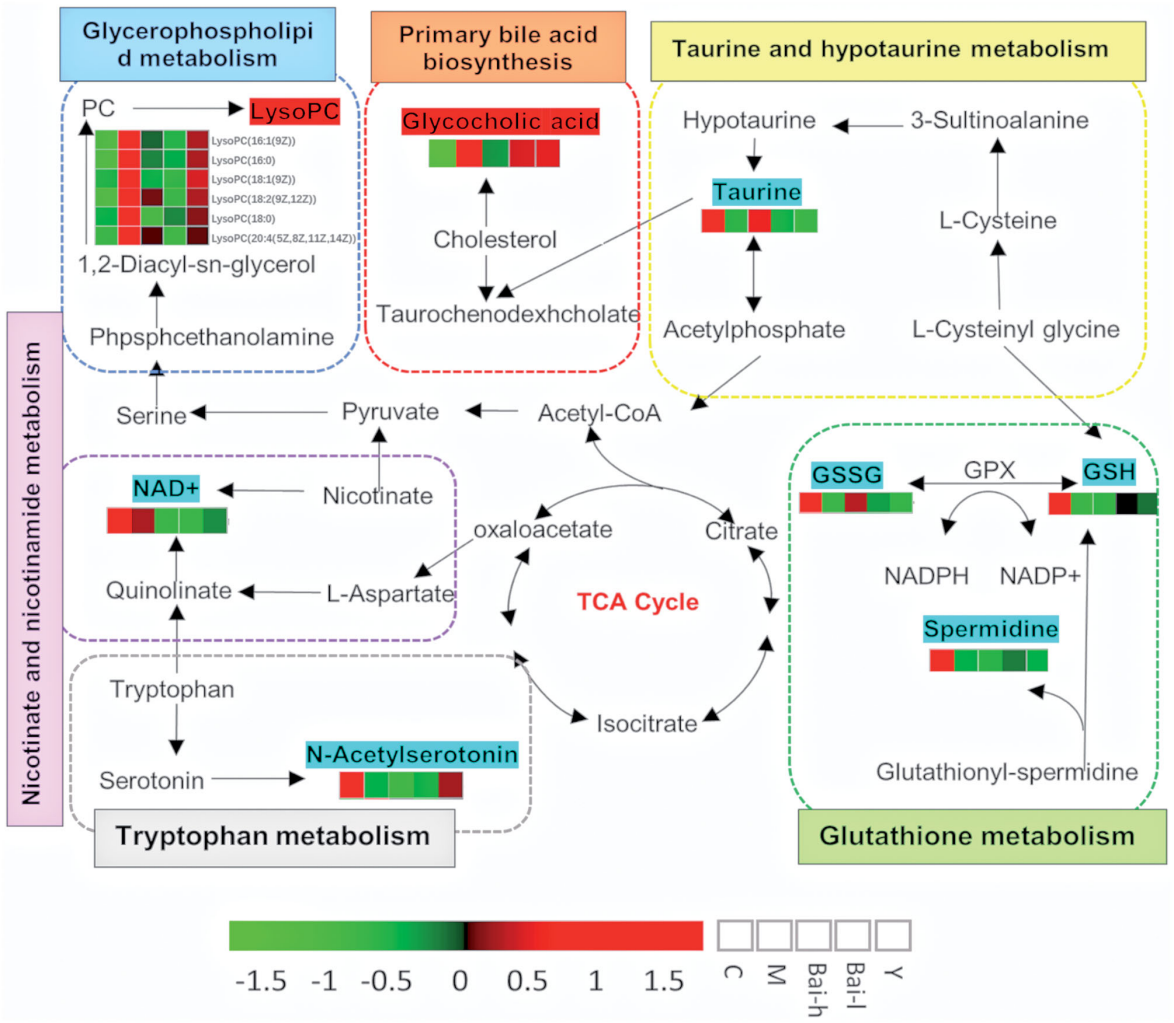
Network of the identified key biomarkers, pathways and the main target of baicalin action according to the KEGG pathway database. The metabolites coloured blue or red represent decreasing or increasing levels, respectively, in the M group compared with the C group.

To clarify and summarise the relationships among these differential metabolites related to PF and their metabolic pathways, we constructed a metabolic network map based on the MetPA results, literature, and KEGG. The results are presented in Figure 5. Revealing the biological significance of these key signalling pathways, their corresponding biomarkers, and their relationship with diseases is of great importance to clarify the mechanisms of PF and to provide valuable information about drug intervention targets.

### Taurine and hypotaurine metabolism

Taurine, present in a free state in the body, is known to have important physiological functions, such as antioxidant and anti-inflammatory effects, which may be able to attenuate lung damage (Men et al. 2010). Previous studies reported that taurine and nicotinic acid decrease the HYP content and ameliorate bleomycin-induced lung fibrosis in mice by inhibiting procollagen gene expression and TGF-β mRNA and protein synthesis. Another study showed that taurine and nicotinic acid could reduce the release of interleukin (IL)-1, IL-6, tumour necrosis factor-α (TNF-α), TGF-β inflammatory factors, and fibroblast cytokines by inhibiting activation of the nuclear factor-κB (NF-κB) pathway (Gurujeyalakshmi et al. 2000). Additional studies have demonstrated that taurine can delay the development of CCl_4_-induced hepatic fibrosis in rats by reducing oxidative stress and inhibiting the activation of hepatic stellate cells (HSCs) (Miyazaki et al. 2005). Correspondingly, the removal of taurine leads to chronic inflammation and tissue injury, which then heals by scar formation, thus promoting fibrosis. Other studies have revealed that metabolic disorders of taurine and subtaurine are present in rats with renal fibrosis, and the urine levels of taurine and subtaurine are decreased significantly (Zhang et al. 2015). As expected, the results of the present study were consistent with those previously published. Serum taurine was significantly reduced in bleomycin-exposed rats, suggesting that taurine and subtaurine metabolism is closely related to the progression of PF. This effect may be related to oxidative stress, inflammatory responses, and the regulation of profibrotic cytokines. In addition, taurine was significantly increased by treatment with high-dose baicalin.

### Glutathione metabolism

Glutathione (GSH), a tripeptide containing a cysteine with a free thiol group, is an important intracellular antioxidant and free radical scavenger that plays an important role in the development of organ fibrosis (Liu et al. 2004). A large number of oxygen-free radicals are produced in injured tissues. GSH scavenges these free radicals and in turn, is oxidized to GSSG (Liu et al. 2004; Liu and Gaston 2010). GSH has significantly decreased in rats with experimental multiple organ fibrosis and in patients with idiopathic PF and cystic fibrosis; treatment with GSH can reduce PF and improve pulmonary function (Tirouvanziam et al. 2006). Studies have revealed that the involvement of GSH in the pathogenesis of PF is closely related to TGF-β, which can reduce the GSH content and impede its ability to scavenge oxygen free radicals, resulting in an accumulation of reactive oxygen species and the stimulation of collagen fibre deposition (Liu and Gaston 2010). Among the many profibrotic factors, TGF-β is most closely related to the development of fibrosis. It can promote the formation of fibrosis in tissues by directly stimulating fibroblasts to synthesize collagen fibres, fibronectin, and proteoglycans, which leads to ECM accumulation while concomitantly inhibiting ECM degradation (Liu et al. 2004).

Our results showed that the levels of GSH, GSSG, and spermidine in the blood of rats with PF were significantly reduced. These markers were restored to the control levels following treatment with baicalin. Furthermore, a previous report indicated that baicalin exerts therapeutic effects in rats with bleomycin-induced PF by regulating the TGF-β1 pathway (Szapiel et al. 1979). Considering that baicalin also affected the oxidative damage indices SOD and MDA, all currently available data support the hypothesis that the ameliorating effect of baicalin is related to oxidative stress and TGF-β regulation.

### Glycerophospholipid metabolism

During glycerophospholipid metabolism, phosphatidylcholines (PCs) can be hydrolyzed to lysophospholipids (Lpcs) by phospholipase A2. Previous reports have indicated that Lpcs can induce fibrosis primarily by promoting fibroblast proliferation, adhesion, migration, and differentiation, as well as epithelial cell apoptosis and oxidative stress responses (Funke et al. 2012; Shea and Tager 2012; Pyne et al. 2013). Consistently, our results showed that the levels of serum Lpcs, including LysoPC (16:0), LysoPC [20:4 (5Z 8Z 11Z 14Z)], and LysoPC (18:0), were significantly increased in the model group, while they were all reduced after treatment with baicalin.

In addition, the levels of nicotinamide adenine dinucleotide (NAD) and *N*-acetyltryptophan were lower and the level of glycocholic acid was higher in the model group than in the control group at 4 weeks. Among them, NAD can be synthesized from nicotinamide via the salvage pathway and from tryptophan via the *de novo* pathway under the catalysis of nicotinamide mononucleotide adenylyltransferase (NMNAT) (Stein and Imai 2012; Burgos 2011). NAD and nicotinamide participate in nicotinate and nicotinamide metabolism, which plays an essential role in maintaining the normal activities of cells to regulate redox reactions, cellular inflammation, and energy metabolism (Yang et al. 2011). *N*-acetyltryptophan and glycocholic acid are involved in tryptophan metabolism and bile acid synthesis, respectively. Previous studies have strongly implicated these two pathways in fibrosis in rat models (Chang et al. 2017). However, the results of our study did not support any significant ameliorating effects of baicalin on NAD, *N*-acetyltryptophan, or glycocholic acid levels.

Our research-based outcomes suggested that these crucial biomarkers are interrelated and interact with each other to form a network that participates in the development of PF. It is worth noting that PF, a risk factor for a more severe progression of COVID-19, is a major factor in the mortality of COVID-19 patients. Moreover, patients recovering from severe COVID-19 disease are still at serious risk of developing PF (Crisan-Dabija et al. 2020). Our research-based outcomes suggested that baicalin can exert an antifibrotic effect by acting on multiple targets and influencing glutathione, taurine, and glycerophospholipid metabolism. Based on network pharmacology research, baicalin may have potential therapeutic effects against COVID-19. Additionally, baicalin and baicalein have been proven to have inhibitory effects on SARS-CoV *in vitro*. At the same time, they alleviate the complications caused by the virus through anti-inflammatory effects and improve the immune response and other functions (Song et al. 2020). Therefore, baicalin is expected to be an effective drug to prevent an excessive fibrotic response in COVID-19 patients. Along with a deeper understanding of the pathogenesis of COVID-19, its exact effects still need to be verified in clinical trials.

## Conclusions

In this study, a serum metabolomics approach based on UPLC-QTOF/MS combined with biochemical and histopathological analyses was used to evaluate the therapeutic effect of baicalin on PF and to elucidate its anti-PF mechanisms by analyzing the impact of baicalin on the levels of potential biomarkers and proteins involved in various metabolic pathways. After baicalin intervention, fibrosis was effectively attenuated, and the metabolic profiles and fluctuating metabolite levels were normalized or partially reversed. Furthermore, eight crucial biomarkers were selected that were closely correlated with the presence of PF; four of these biomarkers, which are involved in taurine and hypotaurine metabolism, glutathione metabolism, and glycerophospholipid metabolism, were significantly restored to normal levels after baicalin treatment. These findings not only provide insight into the mechanisms of fibrotic pathways in bleomycin-induced PF models but also provide an experimental basis for developing baicalin as a clinical treatment for PF.

However, similar to most studies using animal models, the limitations of this study include the need for further studies to validate our results with additional animal models and in clinical studies. Furthermore, future research should focus on exploring the comprehensive mechanism of baicalin at the molecular level by multiple analytical techniques.

## References

[CIT0001] Burgos ES. 2011. NAMPT in regulated NAD biosynthesis and its pivotal role in human metabolism. Curr Med Chem. 18(13):1947–1961. 21517777 10.2174/092986711795590101

[CIT0002] Cai H, Su S, Li Y, Zeng H, Zhu Z, Guo J, Zhu Y, Guo S, Yu L, Qian D, et al. 2018. Protective effects of *Salvia miltiorrhiza* on adenine-induced chronic renal failure by regulating the metabolic profiling and modulating the NADPH oxidase/ROS/ERK and TGF-β/Smad signaling pathways. J Ethnopharmacol. 212:153–165. 29032117 10.1016/j.jep.2017.09.021

[CIT0003] Chang H, Liu Q, Bai WF, Bai YC, Jia XY, Gao C, Liu QL, Shi SL, Zhou HB. 2020. Protective effects of *Amygdalus mongolica* on rats with renal fibrosis based on serum metabolomics. J Ethnopharmacol. 257:112858. 32278030 10.1016/j.jep.2020.112858

[CIT0004] Chang H, Meng HY, Liu SM, Wang Y, Yang XX, Lu F, Wang HY. 2017. Identification of key metabolic changes during liver fibrosis progression in rats using a urine and serum metabolomics approach. Sci Rep. 7(1):11433. 28900168 10.1038/s41598-017-11759-z PMC5595818

[CIT0005] Chen H, Chen Q, Jiang C-M, Shi G-Y, Sui B-W, Zhang W, Yang L-Z, Li Z-Y, Liu L, Su Y-M, et al. 2018. Triptolide suppresses paraquat induced idiopathic pulmonary fibrosis by inhibiting TGFB1-dependent epithelial mesenchymal transition. Toxicol Lett. 284:1–9. 29195901 10.1016/j.toxlet.2017.11.030

[CIT0006] Chen JY, Qiao K, Liu F, Wu B, Xu X, Jiao GQ, Lu RG, Li HX, Zhao J, Huang J, et al. 2020. Lung transplantation as therapeutic option in acute respiratory distress syndrome for coronavirus disease 2019-related pulmonary fibrosis. Chin Med J. 133(12):1390–1396. 32251003 10.1097/CM9.0000000000000839 PMC7339336

[CIT0007] Chitra P, Saiprasad G, Manikandan R, Sudhandiran G. 2013. Berberine attenuates bleomycin induced pulmonary toxicity and fibrosis via suppressing NF-κB dependant TGF-β activation: a biphasic experimental study. Toxicol Lett. 219(2):178–193. 23523906 10.1016/j.toxlet.2013.03.009

[CIT0008] Crisan-Dabija R, Pavel CA, Popa IV, Tarus A, Burlacu A. 2020. “A chain only as strong as its weakest link”: an up-to-date literature review on the bidirectional interaction of pulmonary fibrosis and COVID-19. J Proteome Res. 19(11):4327–4338. 32883081 10.1021/acs.jproteome.0c00387

[CIT0009] Della LV, Cecchettini A, Del Ry S, Morales MA. 2015. Bleomycin in the setting of lung fibrosis induction: from biological mechanisms to counteractions. Pharmacol Res. 97:122–130. 25959210 10.1016/j.phrs.2015.04.012

[CIT0010] Fang J, Wang W, Sun S, Wang Y, Li Q, Lu X, Qiu M, Zhang Y. 2016. Metabolomics study of renal fibrosis and intervention effects of total aglycone extracts of *Scutellaria baicalensis* in unilateral ureteral obstruction rats. J Ethnopharmacol. 192:20–29. 27286917 10.1016/j.jep.2016.06.014

[CIT0011] Funke M, Zhao Z, Xu Y, Chun J, Tager AM. 2012. The lysophosphatidic acid receptor LPA1 promotes epithelial cell apoptosis after lung injury. Am J Respir Cell Mol Biol. 46(3):355–364. 22021336 10.1165/rcmb.2010-0155OC PMC3326436

[CIT0012] George PM, Wells AU, Jenkins RG. 2020. Pulmonary fibrosis and COVID-19: the potential role for antifibrotic therapy. Lancet Respir Med. 8(8):807–815. 32422178 10.1016/S2213-2600(20)30225-3 PMC7228727

[CIT0013] Gurujeyalakshmi G, Wang Y, Giri SN. 2000. Taurine and niacin block lung injury and fibrosis by down-regulating bleomycin-induced activation of transcription nuclear factor-kappaB in mice. J Pharmacol Exp Ther. 293(1):82–90. 10734156

[CIT0014] Hu SF, Wang YQ, Fan YS, Li HC, Wang CY, Zhang JD, Zhang SJ, Han XL, Wen CP. 2015. Lipidomics revealed idiopathic pulmonary fibrosis-induced hepatic lipid disorders corrected with treatment of baicalin in a murine model. AAPS J. 17(3):711–722. 25762447 10.1208/s12248-014-9714-4 PMC4406959

[CIT0015] Huang X, He Y, Chen Y, Wu P, Gui D, Cai H, Chen A, Chen M, Dai C, Yao D, et al. 2016. Baicalin attenuates bleomycin-induced pulmonary fibrosis via adenosine A2a receptor related TGF-β1-induced ERK1/2 signaling pathway. BMC Pulm Med. 16(1):132–143. 27658704 10.1186/s12890-016-0294-1 PMC5034677

[CIT0016] Jo HE, Troy LK, Keir G, Chambers DC, Holland A, Goh N, Wilsher M, Boer S, Moodley Y, Grainge C, et al. 2018. Treatment of idiopathic pulmonary fibrosis in Australia and New Zealand: a position statement from the Thoracic Society of Australia and New Zealand and the Lung Foundation Australia. Respirology. 23:116–139. 28845557 10.1111/resp.13146

[CIT0017] Liu RM, Gaston PKA. 2010. Oxidative stress and glutathione in TGF-beta-mediated fibrogenesis. Free Radic Biol Med. 48(1):1–15. 19800967 10.1016/j.freeradbiomed.2009.09.026 PMC2818240

[CIT0018] Liu RM, Liu Y, Forman HJ, Olman M, Tarpey MM. 2004. Glutathione regulates transforming growth factor-beta-stimulated collagen production in fibroblasts. Am J Physiol Lung Cell Mol Physiol. 286:121–128. 10.1152/ajplung.00231.2003 12959930

[CIT0019] Liu T, Dai W, Li C, Liu F, Chen Y, Weng D, Chen J. 2015. Baicalin alleviates silica-induced lung inflammation and fibrosis by inhibiting the Th17 response in C57BL/6 mice. J Nat Prod. 78(12):3049–3057. 26605988 10.1021/acs.jnatprod.5b00868

[CIT0020] Lu J, Zhong Y, Lin Z, Lin X, Chen Z, Wu X, Wang N, Zhang H, Huang S, Zhu Y, et al. 2017. Baicalin alleviates radiation-induced epithelial-mesenchymal transition of primary type II alveolar epithelial cells via TGF-β and ERK/GSK3β signaling pathways. Biomed Pharmacother. 95:1219–1224. 28931214 10.1016/j.biopha.2017.09.037

[CIT0021] Men X, Han S, Gao J, Cao G, Zhang L, Yu H, Lu H, Pu J. 2010. Taurine protects against lung damage following limb ischemia reperfusion in the rat by attenuating endoplasmic reticulum stress-induced apoptosis. Acta Orthop. 81(2):263–267. 20148646 10.3109/17453671003587085 PMC2895349

[CIT0022] Miyazaki T, Karube M, Matsuzaki Y, Ikegami T, Doy M, Tanaka N, Bouscarel B. 2005. Taurine inhibits oxidative damage and prevents fibrosis in carbon tetrachloride-induced hepatic fibrosis. J Hepatol. 43(1):117–125. 15893842 10.1016/j.jhep.2005.01.033

[CIT0023] Nair AB, Jacob S. 2016. A simple practice guide for dose conversion between animals and human. J Basic Clin Pharm. 7(2):27–31. 27057123 10.4103/0976-0105.177703 PMC4804402

[CIT0024] Pyne NJ, Dubois G, Pyne S. 2013. Role of sphingosine 1-phosphate and lysophosphatidic acid in fibrosis. Biochim Biophys Acta. 1831(1):228–238. 22801038 10.1016/j.bbalip.2012.07.003

[CIT0025] Shea BS, Tager AM. 2012. Role of the lysophospholipid mediators lysophosphatidic acid and sphingosine 1-phosphate in lung fibrosis. Proc Am Thorac Soc. 9(3):102–110. 22802282 10.1513/pats.201201-005AW PMC5455616

[CIT0026] Song J-W, Long J-Y, Xie L, Zhang L-L, Xie Q-X, Chen H-J, Deng M, Li X-F. 2020. Applications, phytochemistry, pharmacological effects, pharmacokinetics, toxicity of *Scutellaria baicalensis* Georgi. and its probably potential therapeutic effects on COVID-19: a review. Chin Med. 15:102–127. 32994803 10.1186/s13020-020-00384-0 PMC7517065

[CIT0027] Song Y-N, Zhang G-B, Lu Y-Y, Chen Q-L, Yang L, Wang Z-T, Liu P, Su S-B. 2016. Huangqi decoction alleviates dimethylnitrosamine-induced liver fibrosis: an analysis of bile acids metabolic mechanism. J Ethnopharmacol. 189:148–156. 27196295 10.1016/j.jep.2016.05.040

[CIT0028] Srour N, Thébaud B. 2015. Mesenchymal stromal cells in animal bleomycin pulmonary fibrosis models: a systematic review. Stem Cells Transl Med. 4(12):1500–1510. 26494779 10.5966/sctm.2015-0121 PMC4675510

[CIT0029] Stein LR, Imai S. 2012. The dynamic regulation of NAD metabolism in mitochondria. Trends Endocrinol Metab. 23(9):420–428. 22819213 10.1016/j.tem.2012.06.005 PMC3683958

[CIT0030] Szapiel SV, Elson NA, Fulmer JD, Hunninghake GW, Crystal RG. 1979. Bleomycin-induced interstitial pulmonary disease in the nude, athymic mouse. Am Rev Respir Dis. 120(4):893–899. 92208 10.1164/arrd.1979.120.4.893

[CIT0031] Tirouvanziam R, Conrad CK, Bottiglieri T, Herzenberg LA, Moss RB, Herzenberg LA. 2006. High-dose oral *N*-acetylcysteine, a glutathione prodrug, modulates inflammation in cystic fibrosis. Proc Natl Acad Sci USA. 103(12):4628–4633. 16537378 10.1073/pnas.0511304103 PMC1450222

[CIT0032] Wang X, Sun H, Zhang A, Sun W, Wang P, Wang Z. 2011. Potential role of metabolomics apporoaches in the area of traditional Chinese medicine: as pillars of the bridge between Chinese and Western medicine. J Pharm Biomed Anal. 55(5):859–868. 21353755 10.1016/j.jpba.2011.01.042

[CIT0033] White ES, Borok Z, Brown KK, Eickelberg O, Guenther A, Jenkins RG, Kolb M, Martinez FJ, Roman J, Sime P. 2016. An American thoracic society official research statement: future directions in lung fibrosis research. Am J Respir Crit Care Med. 193(7):792–800. 27035782 10.1164/rccm.201602-0254ST PMC5440092

[CIT0034] Wu X, Zhi F, Lun W, Deng Q, Zhang W. 2018. Baicalin inhibits PDGF-BB-induced hepatic stellate cell proliferation, apoptosis, invasion, migration and activation via the miR-3595/ACSL4 axis. Int J Mol Med. 41(4):1992–2002. 29393361 10.3892/ijmm.2018.3427 PMC5810201

[CIT0035] Wuyts WA, Agostini C, Antoniou KM, Bouros D, Chambers RC, Cottin V, Egan JJ, Lambrecht BN, Lories R, Parfrey H, et al. 2013. The pathogenesis of pulmonary fibrosis: a moving target. Eur Respir J. 41(5):1207–1218. 23100500 10.1183/09031936.00073012

[CIT0036] Yan H, Huang Z, Bai Q, Sheng Y, Hao Z, Wang Z, Ji L. 2018. Natural product andrographolide alleviated APAP-induced liver fibrosis by activating Nrf2 antioxidant pathway. Toxicology. 396–397:1–12. 10.1016/j.tox.2018.01.007 29355602

[CIT0037] Yang C, Zheng YQ, Dai M. 2011. Research progress on pharmacological action of nicotinamide. J Clin Pulmonary Med. 16:1914–1915.

[CIT0038] Zhang J, Zhang H, Deng X, Zhang N, Liu B, Xin S, Li G, Xu K. 2018a. Baicalin attenuates non-alcoholic steatohepatitis by suppressing key regulators of lipid metabolism, inflammation and fibrosis in mice. Life Sci. 192:46–54. 29158052 10.1016/j.lfs.2017.11.027

[CIT0039] Zhang ZH, Li MH, Liu D, Chen H, Chen DQ, Tan NH, Ma SC, Zhao YY. 2018b. Rhubarb protect against tubulointerstitial fibrosis by inhibiting TGF-β/Smad pathway and improving abnormal metabolome in chronic kidney disease. Front Pharmacol. 9:1029–1042. 30271345 10.3389/fphar.2018.01029 PMC6146043

[CIT0040] Zhang ZH, Wei F, Vaziri ND, Cheng XL, Bai X, Lin RC, Zhao YY. 2015. Metabolomics insights into chronic kidney disease and modulatory effect of rhubarb against tubulointerstitial fibrosis. Sci Rep. 5:14472–14488. 26412413 10.1038/srep14472 PMC4585987

[CIT0041] Zhao Q, Chen XY, Martin C. 2016. *Scutellaria baicalensis*, the golden herb from the garden of Chinese medicinal plants. Sci Bull. 61(18):1391–1398. 10.1007/s11434-016-1136-5 PMC5031759 27730005

